# A platform for generating clinical intelligence from routine health data in hospitals: PICTURE

**DOI:** 10.1016/j.fhj.2025.100492

**Published:** 2025-12-01

**Authors:** Stuart A Bowyer, Eleni Pissaridou, John Booth, Daniel Key, William A Bryant, Ewart Sheldon, Alexandros Zenonos, Rebecca Pope, Harry Hemingway, Andrew Taylor, Neil J Sebire

**Affiliations:** a Data Research, Innovation and Virtual Environments (DRIVE) Unit, Great Ormond Street Hospital, London, UK; b NIHR GOSH Biomedical Research Centre, London, UK; c Department of Surgery and Cancer, Imperial College London, London, UK; d Roche Products Ltd., Hexagon Place, Shire Park, Falcon Way, Welwyn Garden City, UK; e Institute of Health Informatics, Faculty of Population Health Sciences, University College London, London, UK; f Population, Policy and Practice Research and Teaching Department, UCL Great Ormond Street Institute of Child Health, London, UK

## Abstract

The proliferation of electronic health records has led to an increasing volume of routine data about patients, their conditions and clinical interventions. While these data are widely used by clinicians to inform treatment, there is little standardised aggregation for clinical support or operational insights. This research explores a possible approach to redressing this.

We describe a scalable ‘clinical intelligence tool’ allowing researchers and managers to aggregate and analyse routine data in a generalised form, generating on-demand analyses for clinical planning and hospital operations.

We demonstrate a proof-of-concept implementation, called PICTURE. In addition to detailing the architecture of this tool, we show how it can address analytics questions, such as providing an informatics consult to address clinicians’ questions.

We show that aggregating health record data in a clinical intelligence tool has the potential to improve clinical practice, healthcare provider operations and more. The key innovations are the generalisation and standardisation of analytical methods for reuse across a range of EHR data; the use of generic data models to support the reusability and composability of analyses; and the approach of developing analytic functionality with open-source languages and modern software development practices.

## Highlights

• Electronic health records aid patient care and hospital operations, but lack broad analytical platforms.
• This study demonstrates an approach for healthcare providers to easily access clinical data.
• Aggregated data help to answer patient care questions without expert analysts.
• Findings could improve healthcare operations and patient treatment decisions.
• Healthcare providers may rethink electronic records’ role in clinical insights.

**Key messages**

**What is already known on this topic**

The utility of electronic health records in treating individual patients and hospital operations has been widely researched, as have specific analyses and models based on electronic health record data. However, there is little existing evidence concerning the generalised use of a software platform to support the generation of clinical information from electronic health record data.

**What this study adds**

This research has illustrated how it is possible to democratise access to, and use of, aggregated clinical data so that answering questions about what happens to patients is an achievable outcome for a typical healthcare provider with an electronic health record system without requiring researchers or a large team of analysts.

**How this study might affect research, practice or policy**

The clinical information extracted with this method can help healthcare providers provide better care and improve their operations, and has the potential to change the way that all healthcare providers view their health record data and understand their utility in answering important questions.

Alt-text: Unlabelled box

## Introduction

Healthcare providers are increasingly using electronic health record (EHR) systems, containing patient-level data on diagnoses, interventions and outcomes, for clinical care and management.^1^ While EHRs offer key benefits (eg efficiency, effectiveness and satisfaction^2^), they often lack simple methods to aggregate data for secondary analyses. However, there is enormous potential for leveraging these data: such as generating risk scores, comparing rare disease characteristics, forecasting service usage and evaluating post-market medications. Although randomised controlled trials remain the gold standard for clinical evidence, EHR-based observational studies may be more appropriate in certain cases (eg rare diseases or complex comorbidities).

Although multi-centre real-world evidence is well researched,^3^ less attention is given to systematic single-centre approaches, which are critical in settings where external data sharing is limited. Single-centre EHR studies have identified clinical associations^4^ and described patient characteristics.^5^ More recently, ‘informatics consults’^6^^,^^7^ have used in-house EHR data to answer specific questions not addressed by existing literature. Machine learning (ML) models further expand the possibilities for clinical decision support using EHR data.

A key barrier is that EHR data remain largely inaccessible^8^ to most clinicians and researchers. Existing solutions often address narrow questions, rely on ad-hoc methods or use proprietary systems: limiting scalability, reproducibility and interoperability. While business intelligence (BI) tools are common in other industries,^9^^,^^10^ they have seen limited clinical use outside high-level operational monitoring. CogStack^11^ addresses a similar challenge for unstructured text, but focuses less on downstream analytics.

To overcome these challenges, we developed PICTURE (Paediatric Informatics Consultation Using Real-world Evidence), a scalable analytics platform that supports easy, efficient and effective aggregation and analysis of routine health data. Specifically, we aimed to (a) allow users to define arbitrary patient cohorts, (b) provision EHR data for those cohorts, (c) offer extensive analytics functionality, (d) present outputs conveniently, and (e) maintain applicability across various healthcare organisations and settings.

This paper presents our work by detailing three distinct contributions.

First, we describe the proof-of-concept PICTURE prototype implemented at our institution. This platform enables users to define arbitrary patient cohorts, provision routine EHR data for analysis, and apply a library of analytical functions with convenient outputs.

Second, we demonstrate the broader potential of this approach through four diverse example applications, ranging from clinical informatics consults to operational analysis.

Finally, we discuss the underlying methodology and system design as a generalisable architectural pattern. This pattern can be adopted by other healthcare organisations seeking to transform their own routine data into actionable intelligence.

## Methods

**Fig. 1:**
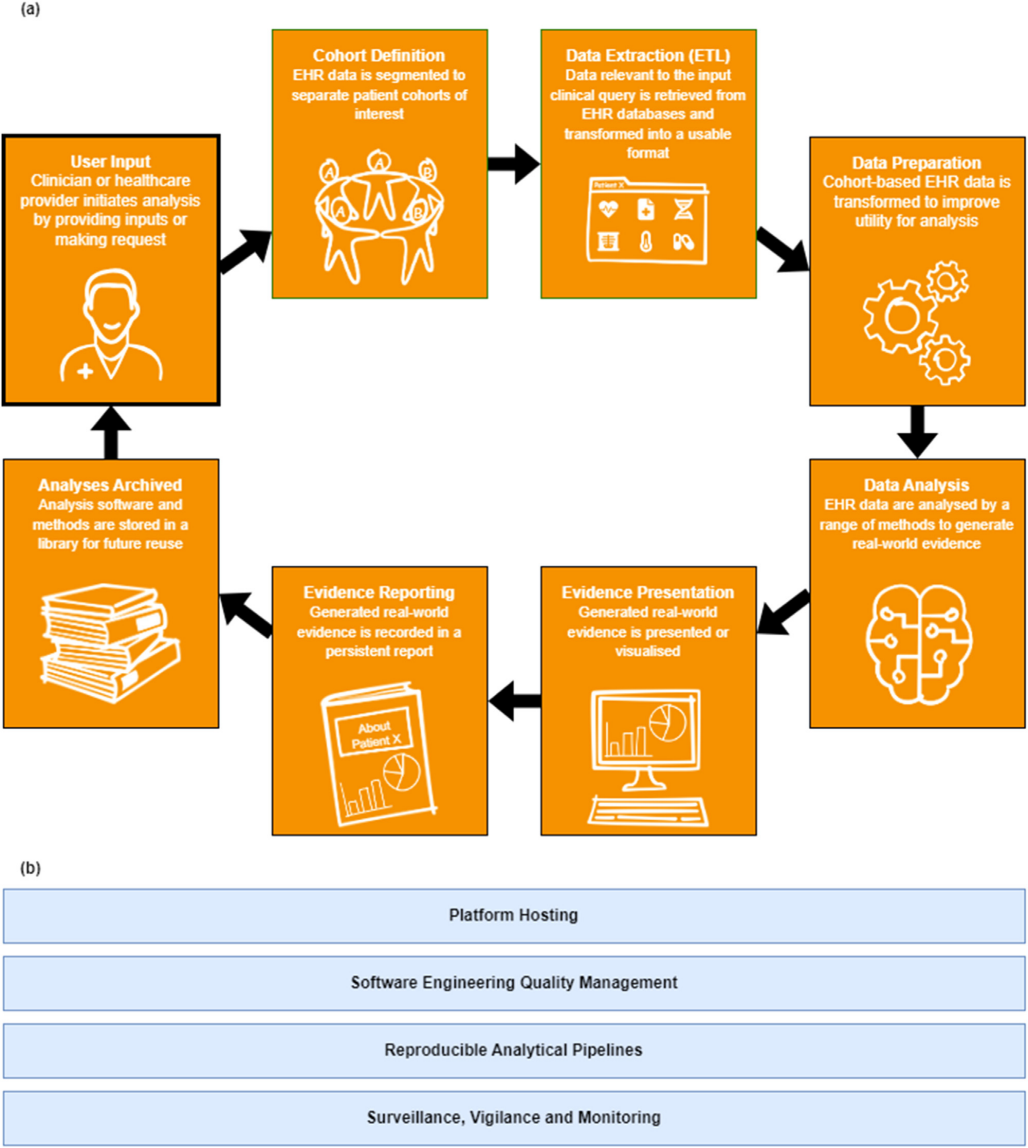
Schematic of the standardised analytics platform involved in the extraction of information from EHR data. (a) Core stages in the analytics pipeline; (b) core platform elements that can support the development, delivery and regulation/governance of platform applications.

**Fig. 2:**
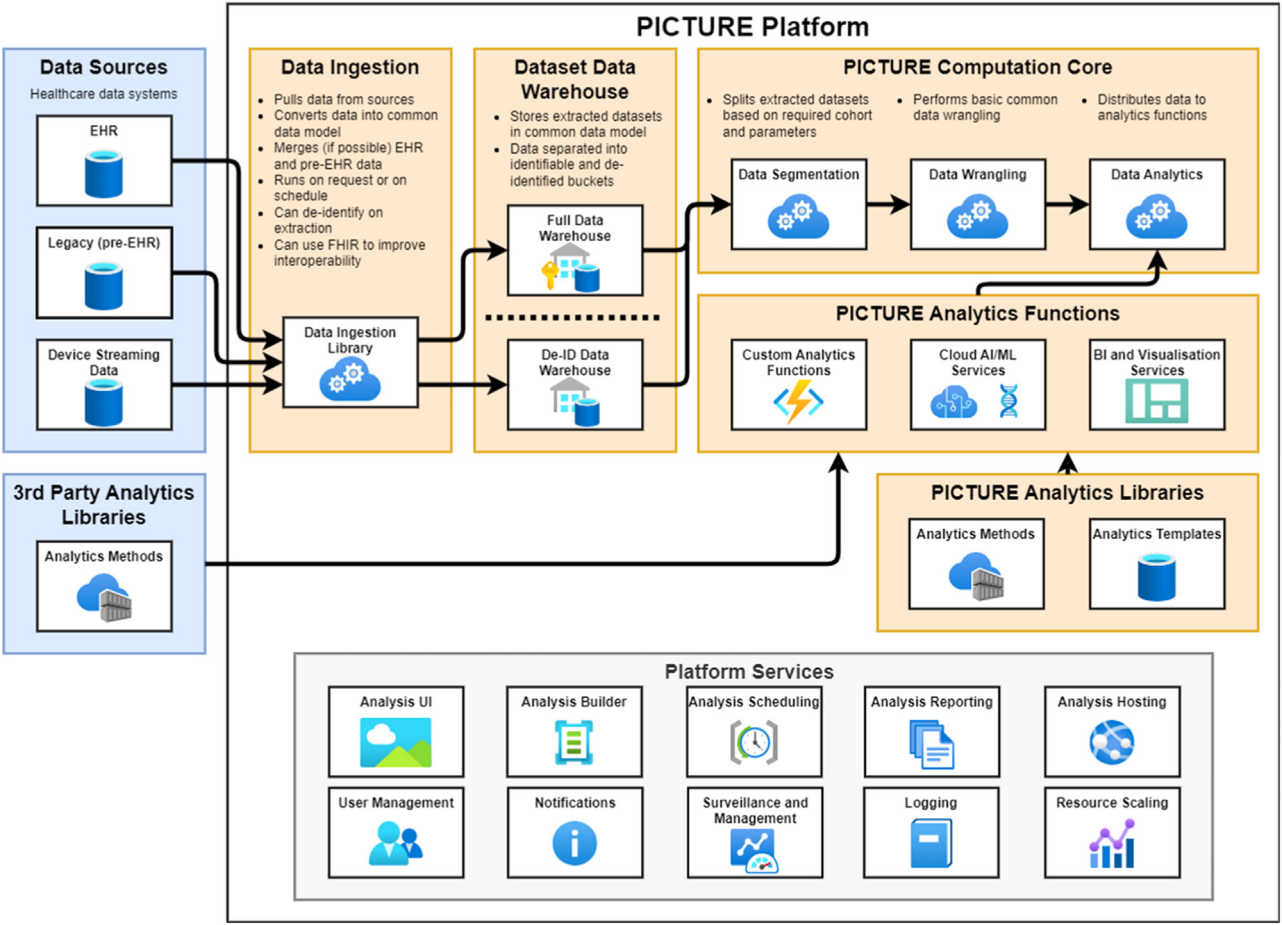
PICTURE schematic showing the simplified architecture. In blue are the platform inputs, orange are the primary platform functions and in yellow are the supporting services.

We explored existing literature on EHR-based clinical intelligence generation to identify the common underlying processes as a basis for the analytics platform (Fig. 1a). All EHR-based methods involve (a) defining a patient cohort, (b) extracting relevant data, (c) preparing and analysing data, and (d) presenting and publishing outputs. To support our aim of making this process ‘easy and efficient’, we have also included a final stage whereby analyses are archived and reused in future applications as part of a cycle. In addition, many methods include other analytics elements that are repeatedly addressed for each application, we have included these as overarching elements (Fig. 1b). Fig. 2 shows a schematic of the PICTURE clinical intelligence platform design, incorporating the standardised analytics stages identified in Fig. 1.

The following methods were developed at the Great Ormond Street Hospital (GOSH). GOSH is a specialist paediatric hospital in London, UK, which sees many patients with rare diseases and complex comorbidities that are not well addressed in the academic literature, such as those through national specialist commissioned services. GOSH was an early UK hospital to deploy a full EHR (Epic) to achieve HIMSS level 7 and has over 600 million structured health data elements in a dedicated NHS trust secure data environment. Structured data are stored with medical classification codes from ICD-10 and SNOMED-CT for diagnoses, OPCS-4 for procedures and GPI for medications.

The platform and its components were developed using open-source languages, primarily R and Python.

All data for analysis and secondary use were de-identified using internal established and approved mechanisms. This involves the removal of all direct identifiers (eg name, NHS number, address) and replacing the internal patient record number with a study-specific pseudonym. Small sample suppression was also employed. To preserve the integrity of longitudinal and temporal analyses, event dates are maintained; data security is instead ensured by performing all analyses within a fully audited workspace, the NHS Trust’s secure data environment, from which no data are permitted to leave. The study was conducted under overarching ethics approval REC 21/LO/0646 for the creation and use of a research database for secondary analysis. The work was carried out within the NHS organisation as part of a joint area of activity that was a component of a wider industry collaboration (with Roche Products Ltd) relating to improved use of data and technology across the NHS. No staff outside the NHS informatics team had access to healthcare data, identifiable or non-identifiable, but industry team members provided input regarding software best practices.

### Cohort and data selection

Generating clinical intelligence from routine EHR content is based on analysing data of a specific patient cohort of interest. The PICTURE approach to defining patient cohorts is based on that described in the OHDSI/OMOP ATLAS,^12^ though other approaches are equally feasible.^13^, ^14^, ^15^ PICTURE incorporates a code-free cohort builder to define a computable phenotype^16^^,^^17^ that identifies a list of patients with the prescribed phenotype. The cohort builder allows users to select features from any data field; however, this could be extended to phenotypes from external libraries, such as the HDR Phenotype Library,^18^ allowing standardised definitions.

### Provisioning of EHR data

After cohorts have been defined, data are provisioned from the hospital EHR data warehouse. While the focus of the PICTURE platform is initially using routine EHR-derived data, the design also facilitates the integration of data from the broader healthcare system including previous administrative databases and device streaming data, for example, providing such data can be delivered via a common data model.

In the PICTURE proof of concept application, data are extracted from the Epic EHR warehouse (Caboodle) and historical administrative databases by the existing research extraction, transform and load (ETL) pipeline.^19^ Data are transformed into a bespoke in-house common data model developed as part of a pre-existing ETL library^19^ that describes clinical events, such as diagnoses and procedures in different tables, in a standardised, reproducible way.

Ingested datasets are stored in PICTURE in the Parquet^20^ file format in a data warehouse to minimise load on source databases.

### Analytics functionality

Fundamental to the analytics functionality of the PICTURE platform is a library of analytics functions that, in combination with the common data model, provide clinical intelligence in response to input queries. The current PICTURE platform includes analytic methods such as frequency analysis (analysing the prevalence of events), measures-of-association analysis (analysing relationships between events) and value-based analysis (analysing distributions in observation values).

Crucially, if analytic functionality is required that does not exist in the library, it can be created using an open-source programming language and integrated with PICTURE. By using a common data model and general structure, it can be easily reused in future applications with similar input data. This design enforces the idea of Reproducible Analytical Pipelines (RAP). RAP is a process by which software engineering best practice is applied to the development of analytics tools and is recommended by the UK government civil service and medical data reviews/guidelines.^21^^,^^22^ PICTURE is designed such that it could incorporate the use of ML/AI analytical services that operate in the cloud or third-party business intelligence visualisation tools.

### Output presentation

The PICTURE platform can render all analytical outputs to an interactive web application and to static output reports, currently provided as PDFs.

### Example applications

To illustrate the utility of the above architecture to address a range of questions, we developed example applications. The current prototype of the PICTURE tool has been developed in the R programming language, making extensive use of the Shiny library^23^ for its front end. The ETL library^19^ has been developed in Python and SQL.

The examples are based on de-identified data from two patient datasets. The nephrology and cardiology datasets contain all patients (*n* = 756 and *n* = 5,033, respectively) who have an episode of care under the nephrology and cardiology specialties, respectively, at the organisation between 1 July 2021 and 30 June 2023. Examples are presented for illustration purposes only to demonstrate aspects of the functionality of the PICTURE tool and are not intended to address further specific clinical or operational issues.

In example 1, we illustrate a clinical informatics consult example for the nephrology dataset exploring how an individual patient’s serum creatinine values compare to those of previous patients who have undergone kidney transplantation surgery, in the period 1 week to 2 years post-surgery. In example 2, we show a patient information example for the nephrology dataset analysing which medications patients undergoing renal haemodialysis typically require. In example 3, we show a research hypothesis generation analysis for the cardiology dataset showing an exploration of the differences between patients who do and do not develop pleural effusion after cardiac surgery. In example 4, we show a clinical operations question for the cardiology dataset showing how soon patients receive their first transthoracic echocardiogram following cardiac defect repair surgery.

## Results

### Example 1: clinical informatics consult for serum creatinine post kidney transplantation

**Fig. 3:**
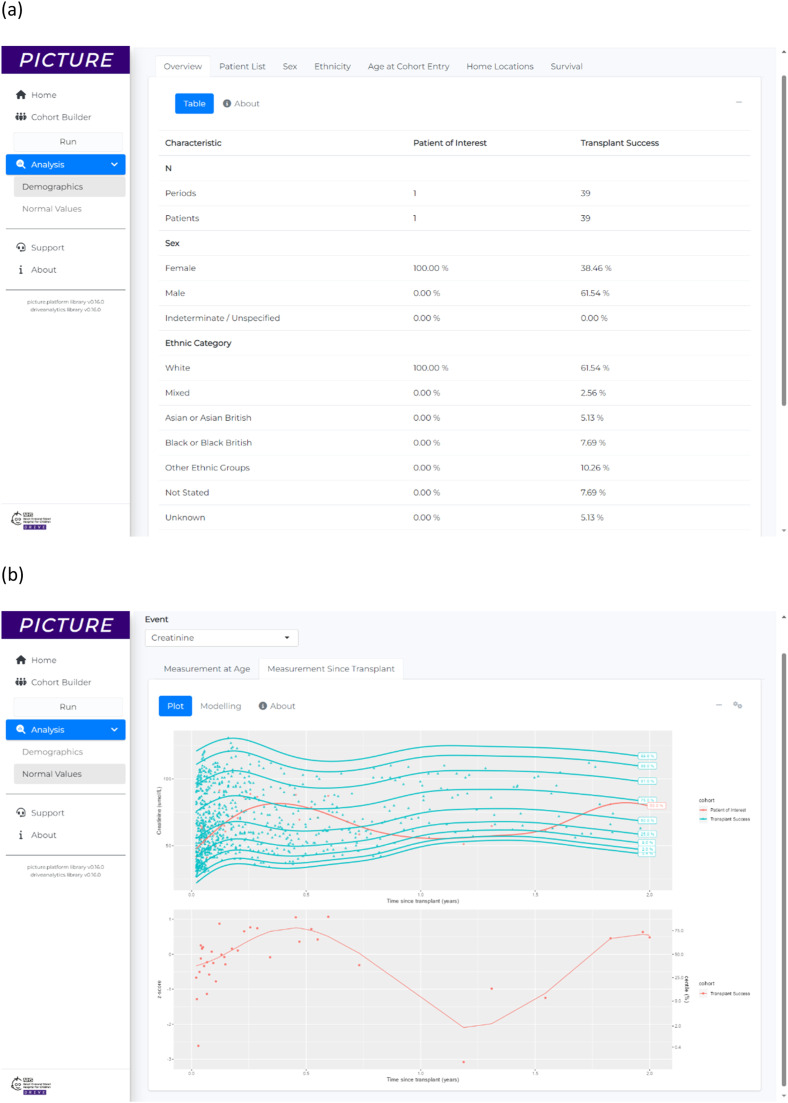
Results for example application 1, showing an informatics consult comparison between a patient of interest and previous successful kidney transplant patients. (a) The demographics overview table showing a summary of the patients from each cohort. (b) A comparison of the serum creatinine values for the patient of interest and previous transplant success patients. The top plot shows a comparison of the raw observed values of serum creatinine for each cohort (coloured points) and the GAMLSS fitted models for each cohort (coloured lines). The bottom plot shows the z-scores and centiles for the patient of interest’s creatinine values compared with the GAMLSS model for the transplant success patients.

The first cohort for this analysis, called ‘Patient of Interest’ (*n* = 1), was defined as an arbitrary anonymous single patient who had undergone OPCS-4 M01 ‘Transplantation of kidney’ procedure. The comparison cohort, called ‘Transplant Success’ (*n* = 39), was defined as all other patients in the dataset who had also undergone OPCS-4 M01 ‘Transplantation of kidney’ procedure and who did not develop subsequent OPCS-4 X40 ‘Compensation for renal failure’ procedure. For both cohorts, their point of entry to the cohort was the date of first OPCS-4 M01 ‘Transplantation of kidney’ procedure. The demographics overview table produced by PICTURE for the analysis is shown in Fig. 3a.

Serum creatinine values were compared using an analytics method that modelled temporal values with Generalised Additive Models for Location, Scale and Shape (GAMLSS). GAMLSS models were fitted to all previous measurements from both cohorts and z-scores were computed from these models to explore whether the ‘Patient of Interest’s serum creatinine values significantly varied from previously observed at the same time point for previous patients. The ‘Transplant Success’ patients were modelled using the Sinh-Arcsinh (SHASH) distribution with P-splines and the ‘Patient of Interest’ values were modelled using a Logistic distribution with P-splines (full details of modelling are beyond the scope of this illustrative overview). Fig. 3b shows the outputs from the modelling component of PICTURE for this analysis showing that ‘Patient of Interest’ had serum creatinine values that were rarely higher than the 75th percentile of the ‘Transplant Success’ cohort.

### Example 2: patient information on medications in renal haemodialysis patients

**Fig. 4:**
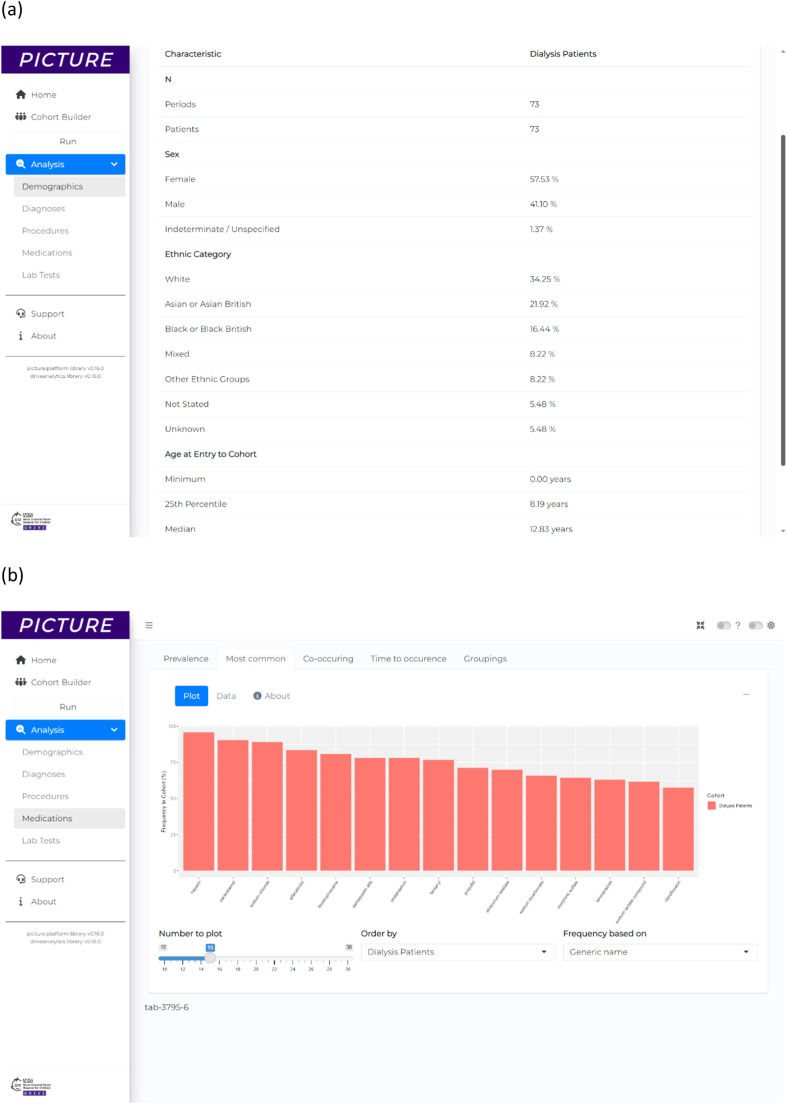

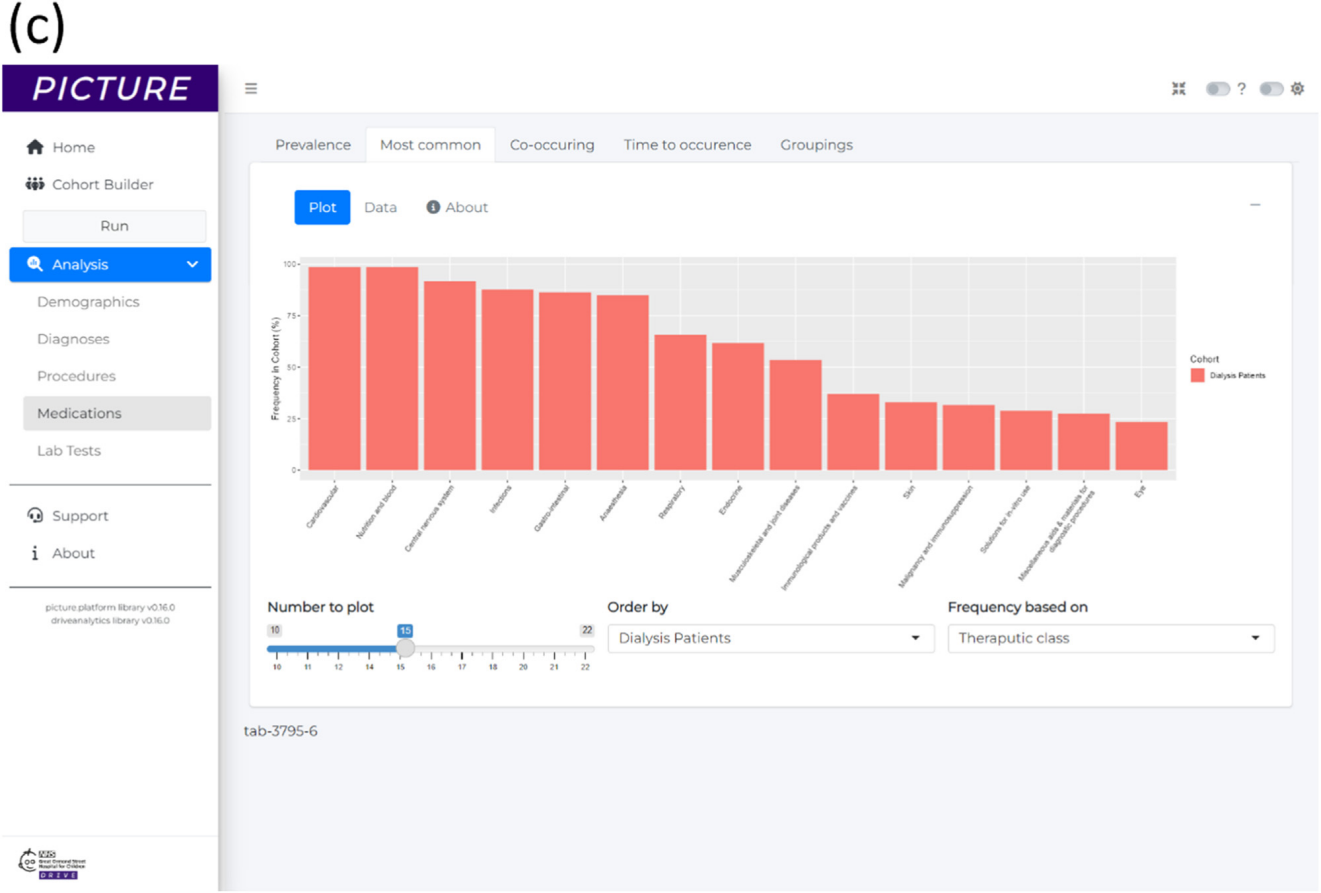
Results for example application 2, showing a generated patient information about the typical medications administered to dialysis patients. (a) The demographics overview table showing a summary of previous dialysis patients. (b) A frequency analysis of the 15 most common medications by generic name. The frequency value is the percentage of patients in the cohort who ever receive the medication at least once after commencing their dialysis. (c) A frequency analysis of the 15 most common medications by therapeutic class.

A cohort was created for this analysis, called ‘Dialysis Patients’ (*n* = 73), containing all patients who had OPCS-4 X40 ‘Compensation for renal failure’ procedure, as well as an ICD-10 N18 ‘Chronic Kidney Disease’ diagnosis with an exclusion criteria for patients who had undergone OPCS-4 M01 ‘Transplantation of kidney’ procedure. The point of entry to the cohort was the date of first OPCS-4 X40 ‘Compensation for renal failure’ procedure. The demographics overview table produced by PICTURE for the analysis is shown in Fig. 4a.

The most common medications that ‘Dialysis Patients’ received were computed using the frequency analysis module, which identified the frequency of all medications for the cohort and plotted the 15 most common. Medications were analysed based both on the ‘Generic Name’ (Fig. 4b) and ‘Therapeutic Class’ (Fig. 4c). The most common generic medication for ‘Dialysis Patients’ is heparin (96%), followed by paracetamol (90%), sodium chloride (89%), alfacalcidol (84%) and levobupivacaine (81%). The analyses also show that over 90% of patients received medications from therapeutic classes ‘Anticoagulants and protamine’, ‘Fluids and electrolytes’, ‘Anaemias and other blood disorders’, ‘Vitamins’ and ‘Analgesics’, for example.

### Example 3: research hypothesis generation in post-cardiac surgery pleural effusion

**Fig. 5:**
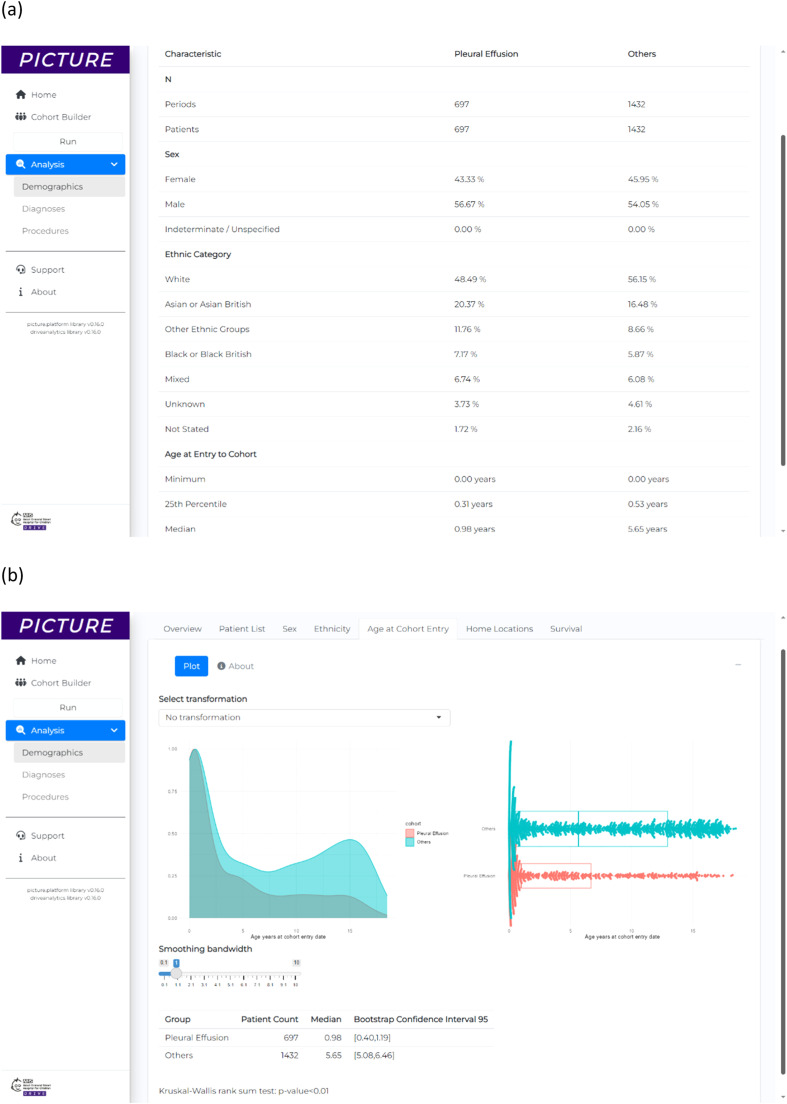

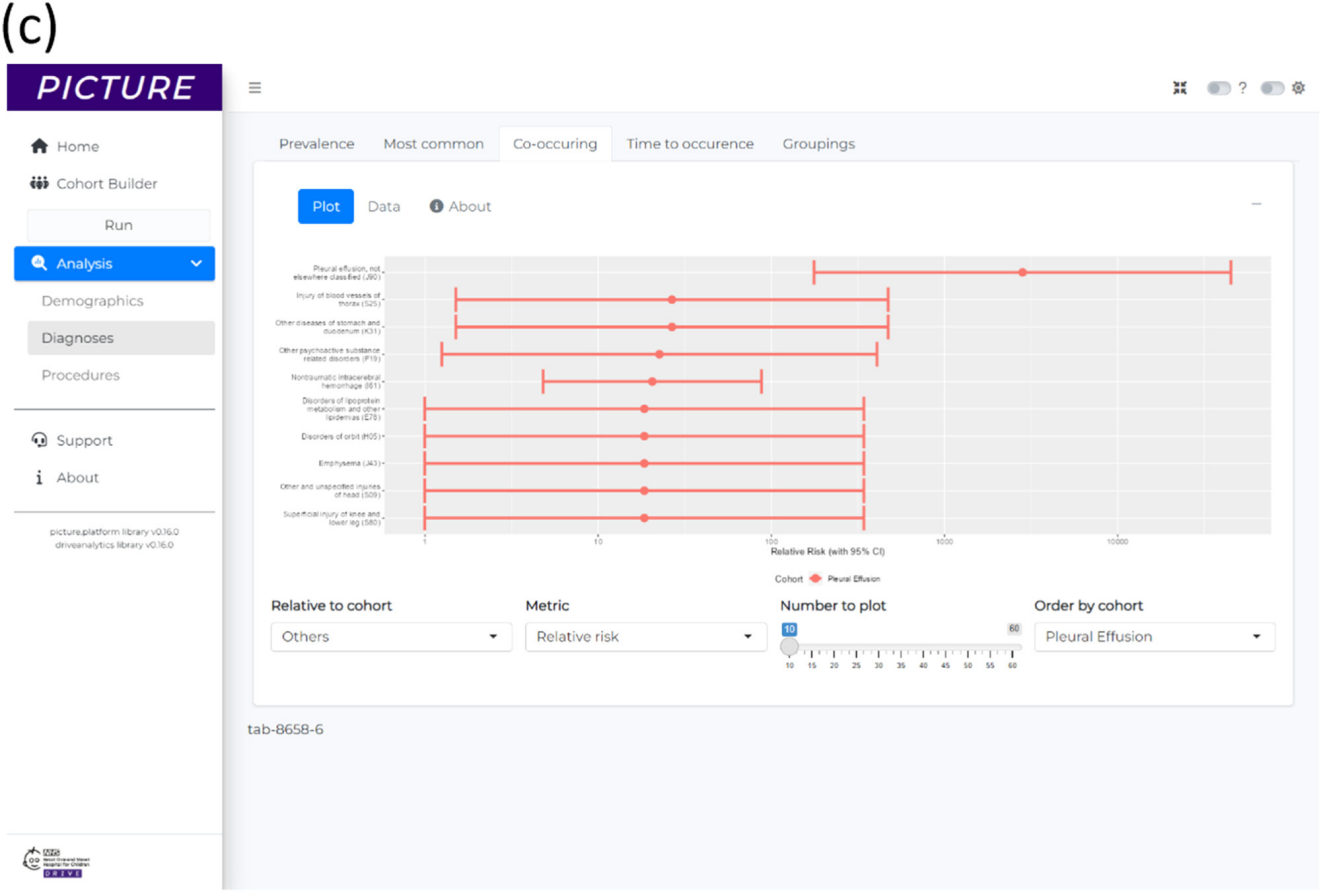
Results for example application 3, showing a research hypothesis generation exploration of patients who develop pleural effusion post-cardiac surgery compared to those who do not. (a) The demographics overview table showing a summary of the patients from each cohort. (b) A value comparison of the age which each cohort had their cardiac surgery, showing the different distributions where the pleural effusion cohort are typically younger than the others. (c) A plot of the 10 highest relative risks for comorbidities in pleural effusion patients compared to the others.

The first cohort for this analysis, ‘Pleural Effusion’ (*n* = 679), was defined as patients from the cardiology dataset who had OPCS-4 K ‘Heart’ procedure and an ICD-10 J90 ‘Pleural effusion, not elsewhere classified’. The second cohort, ‘Others’ (*n* = 1,432), represented patients from the cardiology dataset who had OPCS-4 K ‘Heart’ procedure but never had ICD-10 J90 ‘Pleural effusion, not elsewhere classified’. For both cohorts, their point of entry to the cohort was the date of first OPCS-4 K ‘Heart’ procedure. The demographics overview table produced by PICTURE for the analysis is shown in Fig. 5a.

The age at cohort entry analytics method was used to compare the age at which patients had their first cardiac surgery procedure between cohorts (Fig. 5b). These results show that for both cohorts there is a peak in first cardiac surgery procedures at approximately 1 year of age, but median age for ‘Pleural Effusion’ patients was 0.98 years compared to median age for ‘Other’ of 5.7 years. The plot shows a peak in the ‘Other’ cohort at approximately 15 years of age, that is not visible in the ‘Pleural Effusion’ patients. The Kruskal–Wallis rank sum test showed the ‘Pleural Effusion’ cohort was significantly younger than the ‘Others’ cohort (*p*<0.05).

The relative risk of comorbidities was analysed using the measures of association module of PICTURE that computes and plots relative risk values (Fig. 5c). These findings show that the greatest relative risk diagnoses for the ‘Pleural Effusion’ patients, relative to the ‘Other’ patients (excluding Pleural Effusion itself), are ICD-10 S25 ‘Injury of Blood Vessels of Thorax’ RR = 27 (95% CI 1.5–470), ICD-10 K31 ‘Other diseases of stomach and duodenum’ RR = 27 (95% CI 1.5–470), and ICD-10 F19 ‘Mental and behavioural disorders due to multiple drug use and use of other psychoactive substances’ RR = 23 (95% CI 1.3–410).

### Example 4: operational analysis of transthoracic echocardiogram after interventricular septum defect repair

**Fig. 6:**
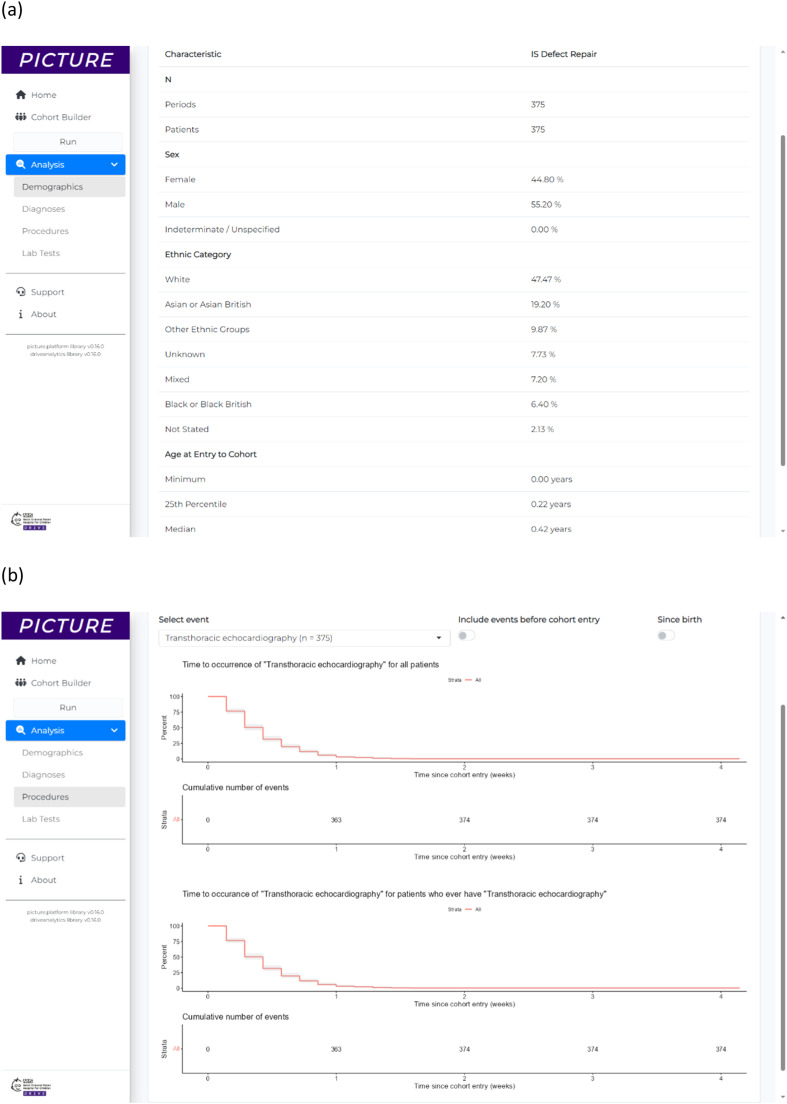
Results for example application 4, showing an operations information analysis exploring the typical requirements for transthoracic echocardiography after interventricular septum defect repair. (a) The demographics overview table showing a summary of the patients from each cohort. (b) A time-to-event analysis showing when during the period 4 weeks after surgery patients typically require a transthoracic echocardiogram.

For this analysis a cohort ‘Defect Repair’ (*n* = 375) was defined as patients from the cardiology dataset with OPCS-4 K11 ‘Repair of defect of interventricular septum’ procedure. Their point of entry to the cohort was the date of first OPCS-4 K11 ‘Repair of defect of interventricular septum’ procedure. The demographics overview table produced by PICTURE for the analysis is shown in Fig. 6a.

Identification of when patients typically undergo transthoracic echocardiography after a defect repair was achieved using the time-to-event analysis module (Fig. 6b). This demonstrated that all but one ‘Defect Repair’ patient had transthoracic echocardiography within 2 weeks of their surgery, and 50% of patients have transthoracic echocardiography performed within 3 days of surgery.

## Discussion

This article demonstrates PICTURE, a platform that embodies a generalisable methodology and architectural pattern that other healthcare organisations can adopt and implement to transform routine EHR data into actionable clinical and operational intelligence. By standardising data extraction, analysis and output, PICTURE addresses diverse use cases – from improving clinical practice and informing patients, to enabling research and supporting hospital operations – within a reproducible software-engineering framework. An important innovation of PICTURE is the provision of a flexible, extensible, but standardised environment in which to provide interactive data exploration and simultaneously support integration of advanced ML and artificial intelligence (AI) tools and arbitrary data and analytics requirements while retaining software engineering best practices.

### A generalisable architectural pattern

The PICTURE platform has been designed to comply with general guidelines and best-practice approaches in the development of clinical informatics, particularly those as set out in the Goldacre review.^22^ By combining aspects from these recommendations, PICTURE presents an example of a significant opportunity for advancing the wider, scalable and reproducible safe secondary use of routine EHR data in healthcare. For analytics developers, the most significant opportunity is that PICTURE supports and enforces use of RAP, as has been recommended by many policy documents and reports.^22^^,^^26^^,^^27^ Specifically, RAP is enforced architecturally. Any new analytical functionality cannot be added in an ad-hoc manner. Instead, it must be developed and submitted to the platform’s analytics library as a self-contained, version-controlled module. This submission process requires the module to have clearly defined inputs and outputs, aligned with the common data model, comprehensive documentation, and a suite of unit tests. All new code undergoes a peer-review process before it can be merged and deployed. This software engineering lifecycle ensures that all analytics within PICTURE adhere to RAP principles. The common data model is fundamental to this, ensuring all analytics are developed against a consistent data structure, which guarantees their reusability across any cohort. This rigorous, expert-led development lifecycle is what enables the key goal of the platform: to allow complex, reproducible analyses to be executed by clinicians and researchers with minimal technical expertise.

### The picture prototype implementation

The generalisable principles described above were used to build the PICTURE prototype, a secure and performant platform operating within the NHS Trust's secure data environment.

The implementation was developed ensuring data security and regulatory compliance. PICTURE, hosted entirely within the NHS organisation’s existing governance framework, with security embedded into its core architecture. By using PICTURE as a centralised platform, security and compliance measures are streamlined, and the risk of noncompliance is mitigated by approved approaches.

The PICTURE architecture is designed for efficient querying of large datasets through its use of the Apache Parquet file format. In practice, for the cohort sizes presented in this work, analyses are typically performed within seconds to a few minutes. However, formal performance benchmarking and load testing with larger cohorts or multiple concurrent users have not been conducted. This remains an important area for future work to ensure the platform can scale effectively as its usage grows.

### Broader potential and future capabilities

Computable phenotype–based ‘informatics consults’ remain a key target for PICTURE. This approach is the subject of a range of research and uses^7^^,^^28^ and has a potential to advance clinical practice by providing on-demand evidence for clinical decision making in cases where guidelines are unavailable or inappropriate. However, translating insight remains a challenge that few have overcome.^29^ The supporting platform and infrastructure of PICTURE can integrate and deploy a wide range of simple informatics consults that improve prospects for short-term translation of usable clinical information.

While currently a single-site implementation, the architectural principles of PICTURE provide a direct pathway to multi-site, federated analytics. In this model, standardised analysis packages could be distributed and executed locally at collaborating institutions, enabling large-scale research without centralising sensitive patient data.

### Challenges and future work

A key consideration for projects such as this is data consistency and quality; with missing data, variability in clinical coding, and changes in practice or measurement standards over time.^24^ To mitigate this, we provide method documentation and data profiling reports, although a quantitative validation of the harmonisation process itself was beyond the scope of this initial work. Future work will develop further checks to ensure valid conclusions and embed statistical controls to prevent misuse and misinterpretation. We will also explore how standardised data preprocessing steps can be embedded in the data pipeline under RAP principles.

A fundamental aspect of unsupervised analytics is the distinction between association and causality. More generally, any unsupervised analytics platform, such as PICTURE, will not be able to immediately address all questions of clinical and operational interest, and specifically questions around inference and causality. Since analyses presented in tools such as this are only able to identify statistical correlations, associations, and predictions, it is generally not possible to identify causality or infer clinical implications or appropriateness of any identified patient group differences since clinical domain expertise is also required. While this presents a challenge in ensuring that users understand the limitations and draw valid conclusions, it is possible for inference analyses to be developed with data extracted and hosted through PICTURE, emphasising the importance of codesign and collaboration between clinical informatics and clinical/operational teams. Further research will also be required to understand the wider implications of the homogeneity of single-centre analyses and clinical management issues that may be specific to a particular organisation.

A primary barrier to scaling PICTURE to other EHRs is the requirement to redevelop the ETL pipeline for each new source system. While the current approach is based on a pre-existing ETL library to extract structured data from the EHR and other clinical operational systems, the diversity of EHR systems and configurations mean that, while using open-source tooling, the system is not currently able to trivially extend to work with additional healthcare providers, which may use different EHR systems and their associated data models and formats.^25^ The future extended use of interoperable standards and open data formats are a potential solution to this issue, and future work is ongoing to explore the potential use of OMOP/FHIR/HL7 to provide cross-organisational interoperability and aggregated dataset analysis.

A further challenge for wider adoption is that while PICTURE is built using open-source languages, the integrated platform codebase is currently proprietary. Therefore, the main contribution of this work is the presentation of a generalisable methodology and architectural pattern that can be replicated, rather than a distributable open-source software package.

A key future priority is to establish a formal patient and public involvement (PPI) group to co-design future platform developments and help define the ethical governance and appropriate uses of the data.

## Conclusions

In conclusion, we have proposed and demonstrated a novel and scalable open approach to the use of routine healthcare data in generating information to support healthcare providers and their patients. PICTURE, the platform that embodies this approach, can easily and efficiently extract information to answer a broad range of questions, and we have demonstrated four diverse examples. With further development and evaluation, this approach has the potential to democratise, promote, and simplify access to and use of aggregated EHR data to improve patient care.

## CRediT authorship contribution statement

**Stuart A Bowyer:** Writing – review & editing, Writing – original draft, Software, Methodology, Conceptualization. **Eleni Pissaridou:** Writing – review & editing, Software, Methodology, Conceptualization. **John Booth:** Writing – review & editing, Software, Data curation. **Daniel Key:** Writing – review & editing, Software, Data curation. **William A Bryant:** Writing – review & editing, Conceptualization. **Ewart Sheldon:** Writing – review & editing, Methodology, Conceptualization. **Alexandros Zenonos:** Writing – review & editing. **Rebecca Pope:** Writing – review & editing. **Harry Hemingway:** Writing – review & editing, Conceptualization. **Andrew Taylor:** Writing – review & editing, Conceptualization. **Neil J Sebire:** Writing – review & editing, Writing – original draft, Supervision, Funding acquisition, Conceptualization.

## Declaration of competing interest

The authors declare the following financial interests/personal relationships which may be considered as potential competing interests: Stuart Bowyer, Eleni Pissaridou, John Booth, Ewart Sheldon reports financial support was provided by Great Ormond Street Hospital Children’s Charity. Stuart Bowyer, Eleni Pissaridou, John Booth, Daniel Key, William Bryant, Ewart Sheldon, Andrew Taylor, Neil Sebire reports financial support was provided by NIHR Great Ormond Street Hospital Biomedical Research Centre. Alexandros Zenonos, Rebecca Pope reports financial support was provided by Roche Products Ltd. If there are other authors, they declare that they have no known competing financial interests or personal relationships that could have appeared to influence the work reported in this paper.
